# *HIF1A:* A Putative Modifier of Hemochromatosis

**DOI:** 10.3390/ijms22031245

**Published:** 2021-01-27

**Authors:** Sara Pelucchi, Giulia Ravasi, Cristina Arosio, Mario Mauri, Rocco Piazza, Raffaella Mariani, Alberto Piperno

**Affiliations:** 1 Department of Medicine and Surgery, University of Milano-Bicocca, 20900 Monza, Italy; sara.pelucchi@unimib.it (S.P.); giulia.ravasi@unimib.it (G.R.); mario.mauri@unimib.it (M.M.); rocco.piazza@unimib.it (R.P.); 2 Liceo Artistico Statale Amedeo Modigliani, 20833 Giussano, Italy; arocri@virgilio.it; 3 Hematology and Clinical Research Unit, ASST-Monza, San Gerardo Hospital Monza, 20900 Monza, Italy; 4 Centre of European Reference Network (EuroBloodNet) and Centre for Rare Diseases-Disorders of Iron Metabolism-ASST-Monza, San Gerardo Hospital Monza, 20900 Monza, Italy; r.mariani@asst-monza.it; 5 Medical Genetics-ASST-Monza, S. Gerardo Hospital Monza, 20900 Monza, Italy

**Keywords:** hereditary hemochromatosis, *HFE*, modifiers, *HIF1A*

## Abstract

HFE-related hereditary hemochromatosis (HH) is characterized by marked phenotypic heterogeneity. Homozygosity for p.C282Y is a low penetrance genotype suggesting that the HFE-HH is a multifactorial disease resulting from a complex interaction involving a major gene defect, genetic background and environmental factors. We performed a targeted NGS-based gene panel to identify new candidate modifiers by using an extreme phenotype sampling study based on serum ferritin and iron removed/age ratio. We found an increased prevalence of the *HIF1A* p.Phe582Ser and p.Ala588Thr variants in patients with a severe iron and clinical phenotype. Accordingly, Huh-7 cells transfected with both variants showed significantly lower *HAMP* promoter activity by luciferase assay. The qRT-PCR assays showed a downregulation of hepcidin and an upregulation of the HIF1A target genes (*VEGF*, *HMOX*, *FUR*, *TMPRSS6*) in cells transfected with the *HIF1A*-P582S vector. We identified mutations in other genes (e.g., Serpina1) that might have some relevance in single cases in aggravating or mitigating disease manifestation. In conclusion, the present study identified *HIF1A* as a possible modifier of the HFE-HH phenotype cooperating with the genetic defect in downregulating hepcidin synthesis. In addition, this study highlights that an NGS-based approach could broaden our knowledge and help in characterizing the genetic complexity of HFE-HH patients with a severe phenotype expression.

## 1. Introduction

Type 1 hemochromatosis (HH) is the most common inherited iron overload disorders in Caucasians, and homozygosity for the p.Cys282Tyr mutation of the *HFE* gene is the most frequent disease-causing genotype. Homozygotes for p.Cys282Tyr may develop iron-related complications, but clinical penetrance is low and expression largely differs between cases. Thus, only a proportion of these subjects will progress to exhibit the full HH phenotype [1]. Acquired factors may influence the HH phenotypes [2,3] and there is evidence that variants in genes involved in hepcidin regulation, iron uptake and transport, and other genes not directly linked to iron metabolism, e.g., *SERPINA1*, *HP*, *GNPAT* and *PCSK7* might modulate the expression of the disease [4,5,6,7,8,9,10,11]. However, none of the polymorphic variants identified to date in candidate-gene and genome-wide association studies emerged as major modifiers of HH phenotype. This supports the idea that type 1 HH is a multifactorial disease resulting from a complex interaction involving a major gene defect, genetic background and environmental factors (e.g., alcohol intake and liver steatosis) [12,13,14]. In the present study, we performed a targeted NGS-based gene panel to identify new candidate polymorphisms that may act as modulators of the disease phenotype in Italian p.Cys282Tyr homozygous patients.

## 2. Results

### 2.1. Iron and Clinical Status

Table 1 shows the main data of the 99 HH patients divided according to the HAS-score (Haute Autorité de Santé) (0–2 vs. 3–4); 41 patients were classified in the less severe group (HAS-low) and 58 in the more severe group (HAS-high). As expected, they significantly differed for sFerr (serum Ferritin), TSAT (Transferrin SATuration) and IR/age (Iron Removed), liver function tests and glycemia. Clinical complications were significantly more frequent in HAS-high than in HAS-low (liver fibrosis in 93.1% vs. 12.1%, of whom 31% vs. 0% had liver cirrhosis; diabetes or glucose intolerance in 27.6% vs. 3%, hypogonadism in 17.2% and cardiopathy in 13.8% vs. none). Table 2 shows the data of the 26 HH patients divided according to extreme phenotypes (L-Iron and H-Iron), selected as reported in the Materials and Methods Section. There were significant differences in iron and hepatic indices, BMI (Body Mass Index) and glucose between patients’ subgroups. None of the patients in the L-Iron subgroup had organ damage while 55% in the H-Iron subgroup had liver cirrhosis (Ishak’s stage 5 or 6) and 45% moderate–severe fibrosis (stage 3 or 4), 45% had clinical and/or laboratory signs of pituitary hypogonadism, 36% had artropathy, 27% diabetes, and 18% early signs of cardiomyopathy (increased interventricular septum thickness and left atrial diameter) [15].

### 2.2. Analysis of NGS Data

We found 679 different variants, of which 77 were in exons, 88 upstream, 122 downstream, and 1 in the splice site region, and 391 were in introns (Supplementary Table S2). Relative to the 77 exonic variants, 73 were already registered with a reference ID: 41 were synonymous variants, 31 were missense, and one was a nucleotide insertion causing a frameshift. Four mutations are still not registered, three were synonymous (*Furin*, *HIF1A* and *TMPRSS6*), and one was a missense mutation in *IREB2*. Table 3 reports the list of the non-synonymous exonic variants and their allele frequencies in patients and Genome Aggregation Database (gnomAD). Six variants showed different distribution in H- and/or L-Iron HH groups compared to gnomAD (*p* < 0.001). In detail, the p.Ser266Asn (rs10455) in *CYBRD1* is a common non-pathogenic variant that was more represented in the L-Iron group; the rs1456427498 in *IREB2* (p.Tyr8fs), reported as a very rare mutation in gnomAD (allele frequency 0.00065), was more represented in both H- and L-Iron groups (one patient in H-Iron and two in L-Iron in the heterozygous state); the rs2958720 in *IREB2* (p.Val159Leu) is a highly frequent variant that was less represented in both H-Iron and L-Iron groups. The rs2235324 in *TMPRSS6* (p.Lys253Glu) is a common non-deleterious polymorphism [16,17] whose minor allele was not represented in the H-Iron group. Two variants in *HIF1A* (p.Phe582Ser (rs11549465, C > T) and p.Ala588Thr (rs11549467, G > A)) were more frequent in the H-Iron group, and they were in complete linkage disequilibrium. In total, nine out of the 11 patients in the H-Iron (82%) compared to 4 of 15 (27%) carried one or the other *HIF1A* variants [18]. The two variants in *HIF1A* were evaluated in the entire cohort of 99 patients by Sanger’s sequencing. Genotypes carrying the rs11549465 minor allele were more common in HAS-high than in the HAS-light group (19/58 (0.33) vs. 5/41 (0.12), respectively, *p* = 0.019) and those carrying the rs11549467 variant were found only in two HAS-high patients. Focusing on the HH-related gene and on genes causing inherited iron diseases, we found only two known synonymous variants in *TFR2* (p.Ala617Ala) and *SLC40A1* (p.Val221Val), and exonic non-synonymous variants in *CP*, *TF*, and *TMPRSS6* in the heterozygous state, respectively. We also found exonic non-synonymous variants in *BMP6, HP* and *Serpina1*, whose proteins might act as possible modulators of hepcidin synthesis and iron overload [19,20,21]. Of the four *CP* variants, one (rs701753, p.Glu544Asp) was a non-pathogenic polymorphism equally distributed between the H- and L-Iron groups. The others showed a different distribution between the two groups: two patients in the L-Iron group carried p.Thr551Ile (rs61733458) and p.Arg793His (rs115552500) variants, respectively. p.Thr551Ile was firstly described in Parkinson’s disease patients [22] and classified as probably pathogenetic in silico (SIFT = 0.0; Polyphen = 1.0) as well as p.Arg793His (SIFT = 0.05; Polyphen = 0.892). p.Pro477Leu (rs35331711) (SIFT = 0.0; Polyphen = 0.984) was found in one patient in the H-Iron group. *TF* variants were common non-deleterious polymorphisms equally distributed between the L- and H-Iron groups. While the common p.Val736Ala variant in *TMPRSS6* was similarly frequent in the H- and L-iron groups, another two polymorphisms (p.Lys235Glu, rs2235324; p.Ala31Val, rs200558933) were identified only in four patients in the L-Iron group. A single patient in the L-Iron group carried the very rare p.Gln114Arg variant in *BMP6* (rs377443730) currently classified as a VUS (variant of uncertain significance). Two patients in the L-Iron group showed two rare variants in *HP* (p.Ala371Ser, rs376612221; p.Val258Leu, rs746679401), classified as deleterious or uncertain, respectively; both patients showed normal serum haptoglobin levels (160 and 132 mg/dL; normal values: 30–200 mg/dL). Lastly, three patients carried mutations in *Serpina1* encoding α1-antitrypsin (A1AT). Two were in the L-Iron group and carried the p.Glu288Val responsible for the PiS form [23]. Accordingly, they had serum A1AT levels in the range of PiS heterozygotes (84 and 82 mg/dL, respectively; normal range 90–200 mg/dL). The other patient belonged to the H-Iron group and carried the p.Glu366Lys, described as PiZ [24], in the homozygous state, and displayed a very low serum A1AT level (25 mg/dL), as expected.

### 2.3. In Vitro Study

**Figure 1:**
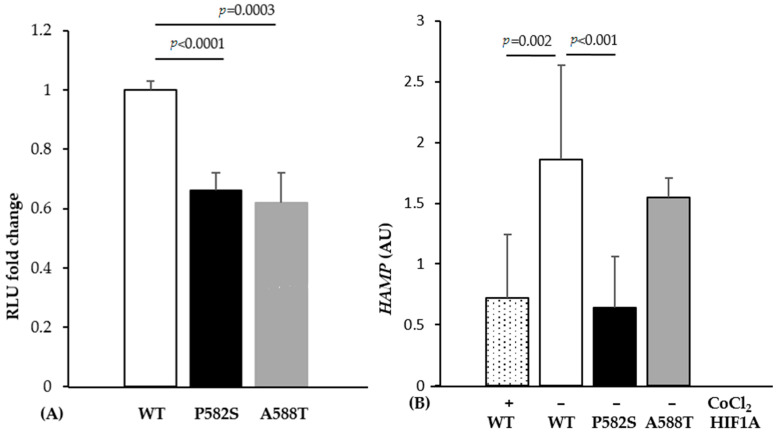
(**A**) Luciferase activity expressed as a fold change in the wild type (WT) and mutant vectors (P582S and A588T), as reported. (**B**) The *HAMP* mRNA levels expressed as the ratio between transfected and non-transfected Huh-7 cells under normoxic (−) and hypoxic (+) conditions. All data were obtained from three independent experiments done in triplicate.

Luciferase assays showed a significant decrease in relative luminescence unit (RLU) when cells were co-transfected with mutant-containing vectors compared to wild type (34% and 38% decrease for HIF1A-P582S and HIF1A-A588T, respectively) (Figure 1A). Cells transfected with the *HIF1A*-P582S, but not with the *HIF1A*-A588T vector, showed a significant downregulation of *HAMP* expression compared to the wild type vector, comparable to that occurring in cells transfected with the *HIF1A*-WT vector treated with CoCl_2_ (Figure 1B).

To confirm the correct transfection, we quantified the mRNAs of *HIF1A*, and genes that are known to be upregulated by the binding of HIF1A on HIF responsive elements (*VEGF*, *HMOX*, *FUR*, *TMPRSS6*). As expected, they were all upregulated when the cells were transfected with the *HIF1A*-P582S but not with *HIF1A*-A588T vectors (Table 4).

**Figure 2:**
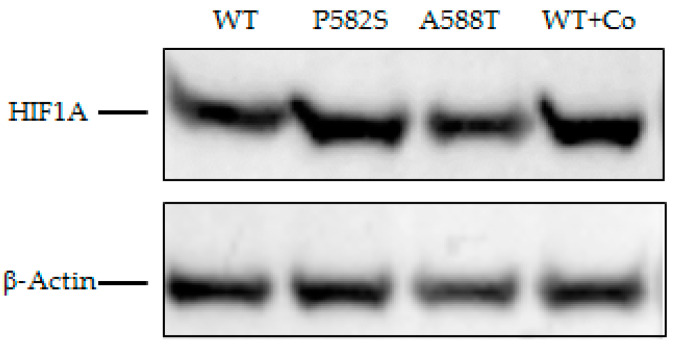
Western blot of lysates of Huh-7 cells transfected with WT vectors in normoxia and chemically induced hypoxia (WT + Co), and with P582S and A588T vectors. β-Actin was used as a loading control.

The Western blot showed increased HIF1A in Huh-7 cell lysates transfected with both variants compared to the HIFA1-wild type. Such an increase was more evident for the *HIF1A*-P582S and comparable with that observed in chemically induced hypoxia cell lysates (Figure 2).

## 3. Discussion

In recent years, several studies aimed at identifying acquired and inherited factors explaining the marked phenotype heterogeneity of HFE-related hemochromatosis (HFE-HH). Gender, age, alcohol intake, and the coexistence of metabolic abnormalities, viral hepatitis and β-thalassemia are all able to influence the severity of iron overload and/or clinical complications [12,14,25]. Supported by studies in the HFE-HH animal model, efforts have been made to identify genetic modifiers in humans [4,5,6,7,8,9,10,25]. Several polymorphic variants in genes involved in iron metabolism or even apparently unrelated (e.g., *PCSK7*, *GNPAT*) have been proposed as potential modifiers of HFE-HH phenotype, although results have often been controversial [9,26]. However, there might be several reasons to explain these differences. First, the phenotypic description of HH remains difficult and imprecise: markers of iron burden are subject to frequent limitations; serum ferritin varies according to age and gender and it can be markedly influenced by several factors that may coexist in HH [12]. Hepatic iron measurement requires liver biopsy that is infrequently performed, or quantitative assessment by magnetic resonance, which is also not routinely done and is less reliable for measuring liver iron concentration in the lower or higher ranges [27]. Indicators of organ damage allow for a precise case description, however, the non-specificity of most clinical symptoms, particularly in less severe cases, and the high prevalence of co-damaging factors, such as metabolic syndrome and alcohol, may result in overestimating the role of iron in the clinical presentation.

In an attempt to overcome these limitations, we exploited the extreme phenotyping sampling study (EPS) for increasing the power of genetic association studies [28]. Although this approach is not free from risks of inflated false positive rates [29], it can be a useful tool in preliminary investigations in complex and heterogeneous disorders.

We firstly selected our cohort for the known cofactors able to influence iron overload and clinical complications. We then sampled patients with an extreme phenotype based on two combined markers of iron burden: serum ferritin and iron removed by phlebotomies corrected for age. Since phlebotomy, with the careful measurement of the amount of iron removed in the blood, is the most accurate measure of total body iron stores [30], we were confident that combining both markers allowed the true selection of two groups with the extreme phenotype, although this reduced the available sample size. Indeed, the two groups markedly and significantly differed both in terms of iron overload and in terms of the severity of the clinical picture (Table 2).

**Figure 3:**
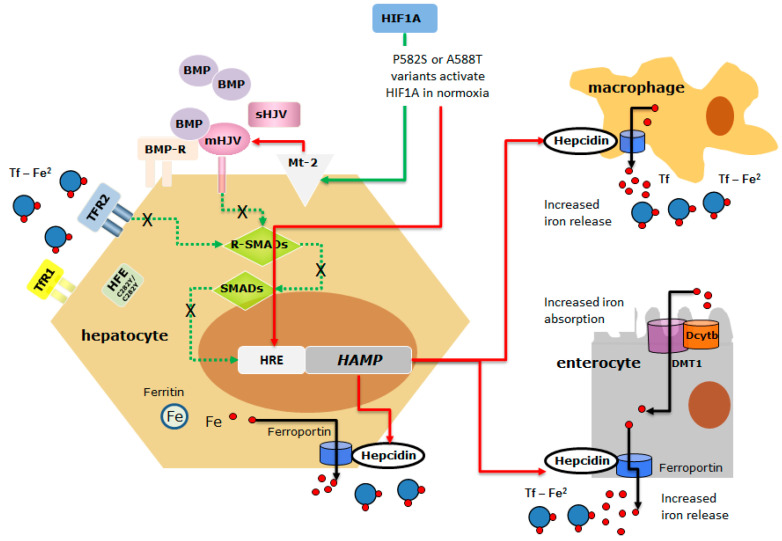
The schematic representation of the possible interaction between *HIF1A* variants and the HFE^C282Y/C282Y^ mutant protein in inducing hepcidin suppression. HFE is a component of an iron-sensing complex that involves interactions with diferric transferrin (Tf-Fe^2^), transferrin receptors (TFR-1 and TFR-2) at the plasma membrane of hepatocytes. High concentrations of Tf–Fe^2^ displace HFE from TFR1, which then forms a complex with TFR2 and HJV to promote bone morphogenetic protein (BMP)/SMAD signaling to hepcidin. HJV is a GPI-linked protein that activates hepcidin as a co-receptor for BMP cytokines. The regulatory SMADs (R-SMADs), SMAD1, SMAD5, and SMAD8 are the mediators of BMP signaling, as they are phosphorylated by activated BMPRs. The common SMAD4 translocates to the nucleus in the complex with the R-SMADs to induce the expression of hepcidin. BMP/SMAD signaling to hepcidin is suppressed by matriptase 2 (Mt-2), a serine protease, codified by *TMPRSS6* which cleaves and generates a soluble form of HJV (sHJV). Homozygous p.Cys282Tyr mutation affects HFE function limiting its expression on the membrane, thus dysregulating the iron-sensing complex and leading to *HAMP* downregulation. HIF1A p.Phe582Ser and p.Ala588Thr variants that stabilize HIF1A in normoxia might further inhibit hepcidin expression acting directly on the *HAMP* Hypoxia Responsive Element (HRE) or indirectly through the activation of Mt-2 [40,49] (the red arrow indicates inhibitory action; the green arrow indicates activating action; dotted green line indicates the signaling pathways dysregulated by HFE^C282Y/C282Y^ mutant protein and HIF-induced Mt-2 activation).

The analysis of the NGS data highlighted three main findings. First, the lack of causal mutations in other HH-related genes confirmed that mutations in non-HFE genes are rare and that di-genic inheritance can occasionally explain the occurrence of a severe phenotype in HFE-HH [31]. Second, we identified six exonic polymorphisms or rare variants whose prevalence differed in patients compared to that reported in gnomAD. However, rs10455 in *CYBRD1* is a common non-pathogenic polymorphism, rs2958720 and rs1456427498 in *IREB2* were under- or over-represented in both L- and H-Iron groups suggesting that they have no relevant role as HFE-HH phenotype modifiers. The rs2235324 in *TMPRSS6* (p.Lys253Glu) is a common polymorphism [16,17] whose minor allele was not found in the H-Iron group. Although the rs2235324 variant in *TMPRSS6* is predicted as being neutral by mutation prediction software [32], two reports from China and Japan suggested that homozygosity for this variant might be implicated in the IRIDA-like phenotype [33,34]. Interestingly, two out of three patients in the L-Iron group carried the rs2235324 variant in the homozygous state, suggesting a possible protective effect on phenotype expression. Thus, the role of this polymorphism remains uncertain and further studies regarding its functional assessment are needed. Due to the increased prevalence of *HIF1A* p.Phe582Ser and p.Ala588Thr variants in H-Iron as well as in HAS-high groups (Haute Autorité de Santé), and their potential role in influencing the stability of HIF1A [18,35], we evaluated their functional role as potential modifiers of the HFE-HH phenotype. There is extensive evidence that the production of hepcidin is downregulated under hypoxic conditions [36,37] and that *HAMP* and other genes involved in hepcidin regulation (*TMPRSS6* and *FUR*) harbor hypoxia-responsive elements (HREs) [38,39]. However, it is controversial whether *HAMP* is a direct target of HIF or if hypoxia-induced hepcidin downregulation occurs indirectly, e.g., through TMPRSS6 upregulation and the increased degradation of membrane-bound hemojuvelin, and/or erythropoiesis activation [37,38,40,41]. Previous studies showed that the HIF1A protein carrying polymorphic p.Phe582Ser or p.Ala588Thr variants displayed higher transactivation capacity in vitro under both normoxic and hypoxic conditions, maintaining the hypoxia-dependent induction response, when compared with the wild type protein [18]. These substituted amino acids are located within or near the N-terminal transactivation domain (N-TAD) and the conserved Pro564 residue, interacting with E3 ubiquitin ligase von Hippel–Lindau tumor suppressor protein (pVHL). It was suggested that one possible mechanism for the observed enhancement of transactivation capacity may be the alteration of protein stability, and that the presence of these variants hinders the hydroxylation of Pro564 residue stabilizing HIF1A and increasing its transcriptional regulation activity in both normoxia and hypoxia [18]. To investigate the role of the two *HIF1A* variants on *HAMP* activity, we performed in vitro experiments on Huh-7, a human hemochromatosis cell line [42]. We showed a comparable reduction in the luciferase activity of the *HAMP* promoter with both variants compared to the wild type. However, the p.Phe582Ser variant seemed stronger than p.Ala588Thr in stabilizing HIF1A activity and inducing *HAMP* downregulation. This is supported by i. the upregulation of the *HIF1A* transcript and protein in cells transfected with the HIF1A-P582S vector; ii. the significant downregulation of *HAMP* mRNA in cells transfected with the HIF1A-P582S vector which was comparable to that observed in chemically induced hypoxia; iii. the significant upregulation of the HIF1A target mRNA genes (*VEGF*, *HMOX*, *FUR*, *TMPRSS6*) in cells transfected with the HIF1A-P582S vector. These findings allow the following conclusions to be made: first, HIF1A can directly modulate the expression of *HAMP* through the binding to the *HAMP* promoter HREs as previously reported [43]; second, the p.Phe582Ser variant mimics a hypoxia status (genetically induced) by stabilizing HIF1A; third, p.Phe582Ser variant *HAMP* inhibition can be the result of the combination of direct (HIF1A-induced) and indirect (the upregulation of *FUR* and *TMPRSS6*) effects [44]. In fact, furin might increase the production of mature hepcidin by pro-hepcidin cleavage, and matriptase-2 may increase the cleavage of hemojuvelin, thus suppressing hepcidin synthesis [40]. Although the rare *HIF1A* p.Ala588Thr (rs11549467) variant was identified exclusively in the more severe H-iron group, its role remains uncertain, as functional studies gave controversial results showing reduced *HAMP*-promoter activity by luciferase assay but no effects on mRNA expression by qRT-PCR assays. Such difference could be due to the higher sensibility of the luciferase assay system to detect even small differences compared to the qRT-PCR [37]. Overall, these results suggest that *HIF1A* variants might be candidate modifiers of the HFE-HH phenotype by aggravating hepcidin suppression, the p.Phe582Ser variant showing higher penetrance than p.Ala588Thr (Figure 3).

A third finding concerns the identification of mutations in *Serpina1* and *Cp* genes that might have some relevance in single cases in aggravating or mitigating disease expression. Six polymorphic variants of α1-antitrypsin, including PiS and PiZ, were identified, whose prevalence did not differ as compared to that in control populations. However, one patient with very severe phenotype (age 40 years, TSAT 88%, s-Ferritin 4728 ng/mL, IR 14.6 g, liver cirrhosis and arthropathy) was also homozygous for the PiZZ allele. This finding reinforces what was observed by Schaefer et al. [45] suggesting that the coinheritance of homozygosity for the PiZZ allele could be a trigger of hepatic iron overload in genetically predisposed individuals by suppressing hepcidin synthesis.

We found four *CP* variants in the L- and H-iron groups, of whom one is a common polymorphism (p.Glu544Asp). Two patients in the L-Iron group carried the p.Thr551Ile variant (rs61733458) and p.Arg793His (rs115552500), respectively. Although described as likely pathogenetic by in silico testing, it is uncertain whether they have any clinical significance (https://web.expasy.org, accessed on 20 December 2020) [46]. Hochstrasser et al. [47] reported a slight reduction in ceruloplasmin concentrations and ferroxidase activity in patients with Parkinson’s disease carrying the p.Arg793His variant. We performed a functional study of p.Thr551Ile variant in HepG2 cells: the ceruloplasmin band was absent in both cell lysate and in culture media, supporting its damaging effect (personal data). The last variant p.Pro477Leu (rs35331711) identified in one patient in the H-iron group seems to have marginal clinical significance (https://clinvarminer.genetics.utah.edu, accessed on 20 December 2020) [48] as also shown by a functional study (personal data). Overall, these findings do not allow for speculation on the role of Cp variants in modulating phenotype expression in patients with HFE-HH. However, further studies may be worthwhile considering the role of the protein in iron recycling.

In conclusion, the present study identified HIF1A as a possible modifier of the HFE-HH phenotype. In addition, it showed that an NGS-based approach could be used to expand our knowledge and help in characterizing the genetic complexity of HFE-HH patients with severe phenotype expression.

## 4. Materials and Methods

Extreme phenotype sampling (EPS) is a popular study design for increasing the power of genetic association studies. Assuming a large cohort with available continuous phenotype data, EPS only involves genotyping individuals in the top and bottom extremes of the phenotype distribution. The rationale for this design is that the phenotypic extremes are enriched for either deleterious or protective variants and so the power to detect genetic effects can be maintained even while genotyping a smaller subset of a larger cohort [28].

### 4.1. Patients

From the entire cohort of p.Cys282Tyr homozygous patients attending the Centre for iron metabolism disorders at the ASST-Monza, San Gerardo Hospital, we excluded females and patients with coexistent chronic liver diseases (autoimmune hepatitis, chronic B or C virus hepatitis, non-alcoholic steatohepatitis), alcohol intake >50 g/day in men and >30 g/day in women [12], a previous history of regular blood donations, concomitant iron loading anemias and those with metabolic syndrome according to previously defined criteria [50]. Inclusion criteria were: p.Cys282Tyr homozygosity, age and serum iron indices (serum iron, transferrin and ferritin), the assessment of iron-related complications at diagnosis and the amount of iron removed based on the number of phlebotomies performed to achieve iron depletion (serum ferritin around 50 ng/mL) [51]. Among the 99 patients fulfilling the exclusion and inclusion criteria, we selected two groups of patients with extreme iron phenotypes, light (L-Iron) and heavy (H-Iron) according to ferritin levels (sFerr) and the amount of iron removed corrected for the age (IR/age) as previously reported [9,52]. The L-Iron group included patients with sFerr ≤ 1000 µg/L and IR/age ≤ 0.15 g/years, while the H-Iron group included patients with sFerr ≥ 2000 µg/L and IR/age ≥ 0.33 g/years. The 26 patients with extreme phenotypes underwent molecular study by the NGS-based panel in order to identify candidate modifiers. Variants that showed significant different allele frequencies in the H-Iron group compared to those in Genome Aggregation Database (gnomAD) [53] were subsequently re-analyzed by Sanger sequencing for confirmation. Two variants in *HIF1A* (rs11549465 and rs11549467) so identified, were analyzed in the whole group of 99 patients according to the HAS-score as previously described [54], and their functional effect on hepcidin expression was evaluated in human hepatic cell lines. All patients gave their written informed consent according to institution’s guidelines.

### 4.2. NGS Panel

A panel of 25 genes and 6 SNPs currently associated with iron metabolism and hereditary iron overload disorders [7,10] was designed and optimized using SureDesign^TM^ software (Agilent Technologies, Santa Clara, CA, USA). The panel consisted of 12.224 amplicons with an average of 200 bp in length, covering a cumulative target sequence of 185.730 Kbp including coding regions, exon/intron junctions, promoters and the target regions of SNPs of interest. The complete list of genes sequenced is reported in Supplementary Table S1.

### 4.3. Library Preparation and Sequencing

Blood samples were collected for DNA extraction from all subjects into tubes containing EDTA. Genomic DNA was extracted from the whole blood of each subject using the Wizard^®^ Genomic DNA Purification kit (Promega, Madison, WI, USA), and stored at −20 °C before use. DNA concentration was measured using the Qubit dsDNA assay kit with Qubit fluorometer 2.0 (ThermoFisher Scientific, Waltham, MA, USA). Custom libraries were generated starting from 225 ng DNA of 26 patients of first cohort using a custom design HaloPlex Target Enrichment kit (Agilent Technologies, Santa Clara, CA, USA) according to the manufacturer’s protocol. Library quality and quantity were determined using the Agilent High Sensitivity DNA kit on the Agilent 2100 bioanalyzer (Agilent Technologies, Santa Clara, CA, USA). Libraries were then sequenced using Ion PGM Hi-Q OT2 kit and Ion PGM Hi-Q Sequencing Kit on Ion PGM 318 chip V2 on an Ion Torrent PGM (ThermoFisher Scientific, Waltham, MA, USA).

### 4.4. Data Analysis

Post run QC/QA filtering was performed using the Torrent Suit (version 3.6; ThermoFisher Scientific, Waltham, MA, USA), and sequences aligned to the human genome version 19 (HG19) using T-MAP (version 3.6.58977, ThermoFisher Scientific, Waltham, MA, USA). Variants were called using the Torrent Variant Caller (version 5.2, with Germ Line–Low Stringency configuration), annotated using wANNOVAR and SureCall^TM^ Software (Agilent Technologies, Santa Clara, CA, USA). The variant calling plugin was set to include variants with a minimum coverage of 20X, following the software developers’ recommendations.

### 4.5. PCR and Sequencing

Direct sequencing of the exon 12 of *HIF1A* was carried out by ABI Prism 3100 Avant DNA sequencer (PE Applied Biosystems, Foster City, CA, USA) for the 73 patients not analyzed by NGS. Sequences were compared with GenBank Accession No. NM_001530. The amplicon of 572 bp covered both rs11549465 (c.1744C > T coding for p.Phe582Ser) and rs11549467 (c.1762G > A coding for p.Ala588Thr).

### 4.6. Mutagenesis

pReceiver-Lv225 vector (Genecopoeia) containing the *HIF1A* wild type gene was mutated into p.Phe582Ser and p.A588T variants using Q5^®^ Site-Directed Mutagenesis Kit (NEB New England BioLabs, Ipswich, MA, USA) according manufacturer’s protocol.

### 4.7. Cell Culture, Transfection and Hypoxia Treatment

The human hepatoma cell line Huh-7 was grown in DMEM (Dulbecco’s modified Eagle medium) supplemented with 10% heat inactivated fetal bovine serum (FBS), glutamine and combined antibiotics, at 37 °C and 5% CO_2_. To examine the effect of HIF1A vectors, cells were seeded in 6-well plates (300,000 cells/well). After 24 h, the cells were transiently transfected with wild type and mutant constructs (1.1 µg) by Fugene HD transfection reagent (Promega, Madison, WI, USA). One day later they were harvested for mRNA isolation and gene expression analysis as reported below. Cells were grown in normoxia and hypoxia, chemically induced with CoCl_2_, as reported in [55]. Experiments were performed in triplicate on three independent occasions.

### 4.8. Dual-Luciferase Reporter Assay

We used a dual-luciferase reporter assay to evaluate the luciferase activity [37]. Huh-7 cells were seeded in 48-well plates at 40% of confluency. Cells were transiently co-transfected with i. the pGL2 reporter vector (Promega Corp., Madison, WI, USA) harboring a 2.9 Kb fragment of human *HAMP* promoter (250 ng) [37]; ii. the pReceiver-Lv225 vector containing the wild type and the mutated (p.Phe582Ser and p.Ala588Thr) *HIF1A* variants, respectively (25 ng/each); iii. the pRL-TK renilla luciferase vector (15 ng) (Promega Corp., Madison, WI, USA) to control transfection efficiency by Fugene HD transfection reagent (Promega Corp., Madison, WI, USA). The pGL-basic plasmids were also co-transfected with the pRL-TK renilla vector as a negative control. After 48 h of transfection, the cells were lysed and firefly and renilla luciferase activities were measured by Glomax Multi JR luminometer according to manufacturer’s protocols (Promega Corp., Madison, WI, USA). Relative luciferase activity was calculated as the ratio between the firefly (reporter) and Renilla luciferase activity and expressed as the RLU fold change compared to wild type. Three independent experiments were performed in triplicate.

### 4.9. RNA Extraction and cDNA Synthesis

RNA from the cell cultures was extracted using the ZR RNA mini-prep (Zymo Research Corporation, Irvine, CA, USA) according to the manufacturer’s protocol, and quantified by spectrophotometry. RNA integrity was assessed by non-denaturing agarose gel. Two hundred and twenty five nanograms of total RNA for a final concentration of 20 ng/µL was used as template for reverse transcription, performed using the High Capacity cDNA Archive kit (Thermo Fisher, Foster City, CA, USA), according to the manufacturer’s protocol.

### 4.10. Real-Time Quantitative-PCR

mRNA expression levels of *HAMP*, *HIF1A*, *VEGF*, *HMOX*, *FUR* and *TMPRSS6* were evaluated by quantitative real-time PCR (qRT-PCR); *HPRT1* was chosen as the housekeeping gene. The analysis was performed on QuantStudio 7 (Thermo Fisher, Foster City, CA, USA) using the Assays-on-Demand Gene Expression Products (Thermo Fisher, Foster City, CA, USA) according to the manufacturer’s protocol. All analyses were carried out in triplicate; results showing a discrepancy greater than 0.3 cycles between the samples were excluded. Relative quantities present in each sample were assessed using the 2^−ΔΔCt^ methods and reported as arbitrary unit (AU). Non-retrotranscribed RNAs were included in each amplification plate, and the analysis was considered valid if the fluorescence intensity in the no-template control was zero.

### 4.11. Western Blot

Proteins obtained from cell lysates were extracted by NET buffer, electrophoresed on NuPAGE 4−12% bis-Tris gel (ThermoFisher Scientific, Waltham, MA, USA) then probed with primary antibodies HIF1α (AbCam, Cambridge, UK) and β-Actin (Sigma, St. Louis, MO, USA) followed by HRP-conjugated secondary antibody (GE Healthcare, Amersham Biosciences Europe GmbH, Freiburg, Germany). Bands were visualized by using enhanced chemiluminescence (GE Healthcare, Amersham Biosciences Europe GmbH, Freiburg, Germany) and images quantified using a Gel-Doc phosphor imager (Bio-Rad Laboratories, Hercules, CA, USA), ImageJ software and normalized by the intensity of β-actin.

### 4.12. Statistical Analysis

Percentages and medians (with first to third quartiles) were used for descriptive purposes, as appropriate. Continuous variables in different groups were compared by non-parametric tests (i.e., Mann–Whitney test). Hardy–Weinberg equilibrium was examined for all subjects using the Chi-squared test. Allele and genotype frequencies in L- and H-iron and HAS-high and -low subgroups were compared using the chi-square or Fisher’s test as needed. The gnomAD data in the European (non-Finnish) populations were used as reference allele frequency. In the in vitro transfection experiments, the *t*-test was used to compare values in normoxia and after hypoxia exposure in wild type and mutant transfected Huh-7 cells. All tests were two sided and with a significance level of α equal to 0.05. Analyses were carried out by the Origin^®^ statistical analysis software (version 2016, OriginLab Corporation, Northampton, MA, USA).

## Figures and Tables

**Table 1:** Biochemical data of 99 patients divided according to the HAS-score.

	All	HAS-Low	HAS-High	*p*
N	99	41	58	//
Age (years)	43 (36–52)	37 (29–45)	45.5 (38.0–58.0)	<0.0001
Hb (g/dL)	15.0 (14.5–15.8)	15.0 (14.7–15.8)	14.9 (13.9–15.6)	ns
sFerritin (µg/L)	1244 (768–2463.5)	765 (516–956)	2164 (1386–3427)	<0.0001
TSAT (%)	84.5 (70.6–90.8)	78.5 (42.0–87.0)	86 (78–95)	<0.0001
IR/age (g/years)	0.15 (0.10–0.24)	0.10 (0.08–0.14)	0.22 (0.14–0.33)	<0.0001
BMI (kg/m^2^)	24.5 (23.5–26.4)	24.4 (23.8–26.6)	24.5 (23.0–26.0)	ns
AST (U/L)	31 (23–44)	25.0 (19.3–30.0)	41 (30–58)	<0.0001
ALT (U/L)	43.0 (26.3–64.8)	27.0 (20.5–45.3)	52.5 (40.0–79.3)	<0.0001
γGT (U/L)	28.5 (18.0–45.3)	18.5 (16.0–30.0)	38.5 (25.0–53.5)	<0.0001
Total cholesterol (mg/dL)	182 (155–212)	172.5 (157.8–207.5)	189 (153–213)	ns
HDL (mg/dL)	49 (42–55)	49.0 (42.5–54.0)	49.0 (42.0–56.5)	ns
Try (mg/dL)	104 (79–144)	92.5 (78.3–127.5)	118 (82–156)	ns
Glu (mg/dL)	93 (85–102)	87.5 (81.8–93.3)	98.5 (89.5–111.3)	0.0001

Data are expressed as median (1st–3rd). Hb: Hemoglobin; TSAT: Transferrin SATuration; IR: Iron Removed; BMI: Body Mass Index; AST: Aspartate Amino Transferase; ALT: Alanine Amino Transferase; γGT: gamma Glutamil Transferase; HDL: High-Density Lipoprotein; Try: Triglycerides; Glu: Glucose; ns: not significant.

**Table 2:** Biochemical data of 26 patients divided according to extreme phenotypes.

	All	L-Iron	H-Iron	*p*
N	26	15	11	//
Age (years)	43 (38–50)	41.0 (37.0–49.5)	43.0 (38.0–48.5)	ns
Hb (g/dL)	14.8 (14.4–15.1)	14.9 (14.6–15.3)	13.9 (12.15–15.75)	ns
sFerritin (µg/L)	1158 (602–3680)	698 (509–794)	4000 (2700–4800)	<0.0001
TSAT (%)	84.5 (64.5–90.0)	70 (59–86)	90 (84–95)	0.01
IR/age (g/years)	0.12 (0.08–0.43)	0.08 (0.07–0.11)	0.46 (0.40–0.63)	<0.0001
BMI (kg/m^2^)	24.2 (23.6–25.3)	24.5 (24.2–26.3)	22.2 (18.8–24.0)	0.008
AST (U/L)	27.0 (21.5–36.0)	23.0 (18.1–27.0)	47.5 (36.0–61.0)	0.0002
ALT (U/L)	38.0 (26.0–50.5)	26 (24–39)	58 (43–86)	0.001
γGT (U/L)	25.0 (16.8–41.5)	19.5 (14.5–28.5)	51.0 (30.5–206.0)	0.009
Total cholesterol (mg/dL)	176.5 (160.3–203.8)	169 (157–183)	205 (189–253)	0.04
HDL (mg/dL)	51.0 (42.0–63.8)	47.5 (42.0–62.5)	57.5 (52.0–65.5)	ns
Try (mg/dL)	98.5 (75.3–145.8)	90 (75–124)	130 (68–170)	ns
Glu (mg/dL)	93 (88–98)	92 (87–95)	105.5 (92.5–151.5)	0.042

Data are expressed as median (1st−3rd). Hb: Hemoglobin; TSAT: Transferrin SATuration; IR: Iron Removed; BMI: Body Mass Index; AST: Aspartate Amino Transferase; ALT: Alanine Amino Transferase; γGT: gamma Glutamil Transferase; HDL: High-Density Lipoprotein; Try: Triglycerides; Glu: Glucose; ns: not significant.

**Table 3:** List of the 41 exonic non-synonymous variants and their allelic frequencies in all 26 patients, subgroups (L-Iron and H-Iron) and Genome Aggregation Database (gnomAD).

Genes	Amino Acid Change	dbSNP	Allelic Frequencies (all)	Allelic Frequencies (L-Iron)	Allelic Frequencies (H-Iron)	Allelic Frequencies (gnomAD)	*p*
ACO1	p.Lys580Gln	rs73477393	0.019	0.000	0.045	0.000	ns
BMP6	p.Gln114Arg	rs377443730	0.019	0.033	0.000	0.000	ns
CP	p.Arg793His	rs115552500	0.019	0.033	0.000	0.009	ns
CP	p.Glu544Asp	rs701753	0.865	0.900	0.818	0.941	ns
CP	p.Pro477Leu	rs35331711	0.019	0.000	0.045	0.004	ns
CP	p.Thr551Ile	rs61733458	0.019	0.033	0.000	0.031	ns
CYBRD1	p.Ser266Asn	rs10455	0.481	0.333	0.682	0.672	<0.0001 *
FAM132B	p.Ala260Ser	rs111241405	0.019	0.000	0.045	0.021	ns
FURIN	p.Arg81Cys	rs148110342	0.019	0.000	0.045	0.002	ns
GNPAT	p.Asp519Gly	rs11558492	0.327	0.300	0.364	0.205	ns
HEPH	p.Val39Ala	rs5919015	0.692	0.733	0.636	0.679	ns
HIF1A	p.Ala588Thr	rs11549467	0.038	0.000	0.091	0.010	0.0001 §
HIF1A	p.Pro582Ser	rs11549465	0.231	0.167	0.318	0.105	0.001 §
HP	p.Ala371Ser	rs376612221	0.019	0.033	0.000	0.000	ns
HP	p.Val258Leu	rs746679401	0.019	0.033	0.000	0.000	ns
IREB2	p.Ile580Thr	rs2230940	0.846	0.733	1.000	0.9998	ns
IREB2	p.Val159Leu	rs2958720	0.500	0.467	0.545	0.9998	<0.0001 *
IREB2	p.Tyr8fs	rs1456427498	0.058	0.067	0.045	0.0001	<0.0001 * < 0.0001 §
SCARA5	p.Ala304Val	rs118119884	0.019	0.000	0.045	0.036	ns
SCARA5	p.Asp316His	rs17058207	0.038	0.067	0.000	0.102	ns
SERPINA1	p.Arg125His	rs709932	0.135	0.133	0.136	0.163	ns
SERPINA1	p.Glu288Val	rs17580	0.038	0.067	0.000	0.037	ns
SERPINA1	p.Glu366Lys	rs28929474	0.038	0.000	0.091	0.018	ns
SERPINA1	p.Glu400Asp	rs1303	0.212	0.167	0.273	0.255	ns
SERPINA1	p.Ile116Met	rs759135389	0.019	0.033	0.000	0.0000	ns
SERPINA1	p.Val237Ala	rs6647	0.173	0.100	0.273	0.217	ns
TF	p.Ile448Val	rs2692696	1.000	1.000	1.000	1.000	ns
TF	p.Pro589Ser	rs1049296	0.135	0.167	0.091	0.160	ns
TFRC	p.Gly142Ser	rs3817672	0.635	0.633	0.636	0.551	ns
TMPRSS6	p.Ala31Val	rs200558933	0.019	0.033	0.000	0.000	ns
TMPRSS6	p.Lys253Glu	rs2235324	0.096	0.167	0.000	0.385	<0.0001 §
TMPRSS6	p.Val736Ala	rs855791	0.442	0.367	0.545	0.564	ns
FURIN	p.Leu163Leu	//	0.019	0.000	0.045		ns
HIF1A	p.Tyr659Tyr	//	0.019	0.000	0.045		ns
IREB2	p.Pro145Ser	//	0.019	0.000	0.045		ns
TMPRSS6	p.Leu746Leu	//	0.019	0.033	0.000		ns

*: L-Iron vs. gnomAD; §: H-Iron vs. gnomAD. ns: not significant.

**Table 4:** *HIF1A*, *VEGF*, *HMOX*, *FUR* and *TMPRSS6* mRNA levels expressed as the ratio between transfected and non-transfected Huh-7 cells under normoxic and hypoxic (+) conditions. All data were obtained from three independent experiments conducted in triplicate (mean ± SD).

	WT + Co	WT	P582S	A588T
**HIF1A**	911 ± 21 *	600 ± 113	20,653 ± 2505 *	287 ± 82
**VEGF**	2.93 ± 0.86 *	0.76 ± 0.05	89 ± 5 *	0.20 ± 0.07
**HMOX**	69 ± 9 *	1.44 ± 0.11	6.28 ± 1.66 *	0.42 ± 0.06
**FUR**	0.63 ± 0.15	0.43 ± 0.10	55 ± 2 *	0.35 ± 0.07
**TMPRSS6**	0.38 ± 0.04	1.06 ± 0.11	77 ± 12 *	0.06 ± 0.02

*: *p* < 0.05 vs. WT.

## Data Availability

Not applicable.

## Supplementary Materials

Supplementary materials can be found at https://www.mdpi.com/1422-0067/22/3/1245/s1.

## Abbreviations

A1AT	Alpha 1 Anti-Trypsin
ALT	Alanine Amino Transferase
AST	Aspartate Amino Transferase
AU	Arbitrary Unit
BMI	Body Mass Index
DMEM	Dulbecco’s Modified Eagle Medium
EPS	Extreme Phenotype Sampling
FBS	Fetal Bovine Serum
gnomAD	Genome Aggregation Database
HAS	Haute Autorité de Santé
HIF1A-WT	Vector Carrying cDNA of *HIF1A* Wild Type
HIF1A-P582S	Vector Carrying cDNA of *HIF1A* with p.Phe582Ser Variant
HIF1A-A588T	Vector Carrying cDNA of *HIF1A* with p.Ala588Thr Variant
HRE	Hypoxia Responsive Element
HDL	High-Density Lipoprotein
HH	Hereditary Hemochromatosis
IR	Iron Removed
NGS	Next Generation Sequencing
qRT-PCR	Quantitative-Real Time Polymerase Chain Reaction
RLU	Relative Luminescence Unit
sFerr	Serum Ferritin
SNP	Single Nucleotide Polymorphism
Try	Triglicerydes
TSAT	Transferrin Saturation
VUS	Variant of Uncertain Significance
