# Supplementary material for: HIF1A: A Putative Modifier of Hemochromatosis
Source: Int J Mol Sci. 2021 Jan 27;22(3):1245. doi: 10.3390/ijms22031245 (PMC7865586; doi:10.3390/ijms22031245)
Supplement: Supplementary file 1 [file ijms-22-01245-s001.zip › SupplTable1ok.docx]

| GENE/SNPs | LOCALIZATION | ROLE IN IRON HOMEOSTASIS |
| --- | --- | --- |
| Aconitase 1 (ACO1, IRP1) | 9p21.1 | Iron sensing |
| Beta-2-microglobulin (B2M) | 15q21.1 | HFE interaction |
| Bone morphogenetic protein 6 (BMP6) | 6p24.3 | Hepcidin regulator |
| Ceruloplasmin (CP) | 3q24-q25.1 | Ferroxidase |
| Cytocrome b reductase 1 (CYBRD1, DCYTB) | 3q24-q25.1 | Ferric reductase |
| Erytroferrone (ERFE, FAM132B) | 2q37.3 | Iron storage |
| Ferritin, heavy polypeptide 1 (FTH1) | 11q12.3 | Iron storage |
| Ferritin, light polypetide (FTL) | 19q13.33 | Iron storage |
| Furin (FURIN) | 15q26.1 | Hepcidin cleaving enzyme (activator) |
| Hepcidin (HAMP) | 19q13.1 | Master regulator |
| Hephaestin (HEPH) | Xq12 | Ferroxidase |
| HFE | 6p22.2 | Hepcidin regulator: iron sensing (HH type 1) |
| Hemojuvelin (HFE2) | 1q21.1 | Hepcidin regulator (HH type 2) |
| Hypoxia inducible factor 1 alfa (HIF1A) | 14q23.3 | Hypoxia sensing |
| Haptoglobin (HP) | 16q22.2 | Hemoglobin transporter |
| Iron-responsive element binding protein 2 (IREB2) | 15q25.1 | Iron sensing |
| Neogenin (NEO1) | 15q24.1 | Hepcidin regulator |
| Scavenger receptor class A member 5 (SCARA5) | 8p21.1 | Ferritin receptor |
| Serpin family member 1 (SERPINA 1) | 14q32.13 | TMPRSS6 inhibitor |
| Solute carrier family 11 member 2 (SLC11A2, DMT1) | 12q13.12 | Iron import |
| Solute carrier family 40 member 1 (SLC40A1, FPN1) | 2q32.2 | Iron export (HH type 4) |
| Transferrin (TF) | 3q22.1 | Iron transport |
| Transferrin receptor (TFRC) | 3q29 | Iron import |
| Transferrin receptor 2 (TFR2) | 7q22.1 | Hepcidin regulator (HH type 3) |
| Transmembrane protease serine 6 (TMPRSS6) | 22q12.3 | Hepcidin regulator (IRIDA) |
| rs651007 (ABO) | 9q34.2 | Putative HH Modifier |
| rs6486121 (ARNTL) | 11p15.3 | Putative HH Modifier |
| rs174577 (FADS2) | 11q12.2 | Putative HH Modifier |
| rs11558492 (GNPAT) | 1q42.2 | Putative HH Modifier |
| rs4921915 (NAT2) | 8p22 | Putative HH Modifier |
| rs236918 (PCSK7) | 11q23.3 | Putative HH Modifier |

**Supplementary Table 1.** List of 25 genes and 6 SNPs analized by NGS, gene localization and role in iron homeostasis.
