# Supplementary material for: HIF1A: A Putative Modifier of Hemochromatosis
Source: Int J Mol Sci. 2021 Jan 27;22(3):1245. doi: 10.3390/ijms22031245 (PMC7865586; doi:10.3390/ijms22031245)
Supplement: Supplementary file 1 [file ijms-22-01245-s001.zip › SupplTable2.docx]

| GENE | UPSTREAM | EXON | INTRON | SPLICESITES | DOWNSTREAM |  |
| --- | --- | --- | --- | --- | --- | --- |
|  | **& 5’_UTR** |  |  |  | **& 3’_UTR** | **Total** |
| Aconitase 1 (ACO1, IRP1) | 1 | 2 | 29 | 0 | 7 | 39 |
| Beta-2-microglobulin (B2M) | 0 | 0 | 1 | 0 | 2 | 3 |
| Bone morphogenetic protein 6 (BMP6) | 2 | 5 | 7 | 0 | 6 | 20 |
| Ceruloplasmin (CP) | 3 | 6 | 28 | 0 | 4 | 41 |
| Cytocrome b reductase 1 (CYBRD1, DCYTB) | 33 | 1 | 17 | 0 | 16 | 67 |
| Erytroferrone (ERFE, FAM132B) | 0 | 1 | 8 | 0 | 6 | 15 |
| Ferritin, heavy polypeptide 1 (FTH1) | 0 | 0 | 0 | 0 | 5 | 5 |
| Ferritin, light polypetide (FTL) | 0 | 1 | 1 | 0 | 1 | 3 |
| Furin (FURIN) | 6 | 6 | 29 | 0 | 4 | 45 |
| [Glyceronephosphate O-acyltransferase](https://www.ncbi.nlm.nih.gov/gene/8443) (GNPAT, rs11558492) | 0 | 1 | 0 | 0 | 0 | 1 |
| Hepcidin (HAMP) | 5 | 0 | 0 | 0 | 0 | 5 |
| Hephaestin (HEPH) | 0 | 3 | 12 | 0 | 3 | 18 |
| HFE | 6 | 0 | 1 | 0 | 4 | 11 |
| Hemojuvelin (HFE2) | 0 | 0 | 1 | 0 | 0 | 1 |
| Hypoxia inducible factor 1 alfa (HIF1A) | 1 | 5 | 9 | 0 | 2 | 17 |
| Haptoglobin (HP) | 0 | 4 | 4 | 0 | 0 | 8 |
| Iron-responsive element binding protein 2 (IREB2) | 1 | 5 | 17 | 0 | 11 | 34 |
| Neogenin (NEO1) | 0 | 3 | 22 | 0 | 6 | 31 |
| Scavenger receptor class A member 5 (SCARA5) | 4 | 7 | 26 | 0 | 14 | 51 |
| Serpin family member 1 (SERPINA 1) | 2 | 6 | 9 | 0 | 11 | 28 |
| Solute carrier family 11 member 2 (SLC11A2, DMT1) | 0 | 1 | 33 | 0 | 8 | 42 |
| Solute carrier family 40 member 1 (SLC40A1, FPN1) | 11 | 1 | 6 | 0 | 3 | 21 |
| Transferrin (TF) | 6 | 6 | 44 | 0 | 0 | 56 |
| Transferrin receptor (TFRC) | 5 | 2 | 36 | 0 | 8 | 51 |
| Transferrin receptor 2 (TFR2) | 0 | 1 | 15 | 0 | 1 | 17 |
| Transmembrane protease serine 6 (TMPRSS6) | 2 | 10 | 36 | 1 | 0 | 49 |
| Total | 88 | 77 | 391 | 1 | 122 |  |

**Supplementary Table 2.** Count of 679 variants found in 25 genes and GNPAT SNP.
